# A systematic review of the patient reported outcome measures used to assess the impact of periodontitis and peri-implantitis on oral health related quality of life

**DOI:** 10.1038/s41405-024-00273-w

**Published:** 2025-03-28

**Authors:** Joshua M. Jackson, Richard Holliday, James S. Hyde, Helen J. Rogers

**Affiliations:** 1 School of Dental Sciences, Faculty of Medical Sciences, Newcastle University, Newcastle upon Tyne, UK; 2 Newcastle Upon Tyne Hospitals Foundation Trust, Newcastle Upon Tyne, UK; 3 School of Dentistry, Cardiff University, Cardiff, UK

**Keywords:** Periodontitis, Gum disease

## Abstract

**Introduction:**

Periodontal disease (PD) and peri-implantitis (PI) can have a significant impact on oral health related quality of life. It is important that any patient reported outcome measures accurately reflect this impact.

**Aims:**

To identify the measures used to assess the impact of periodontal disease and peri-implantitis on oral health related quality of life. To assess the psychometric properties of the measures identified by applying an adapted version of the Consensus-based standards for the selection of health measurement instruments (COSMIN) checklist.

**Materials and methods:**

A search of the literature incorporated relevant MeSH terms across four separate databases: Scopus, Web of Science, Medline and Embase. Following the removal of duplicates, studies meeting the inclusion criteria were screened. Any condition specific measure or a measure appearing greater than five times was analysed in accordance with the COSMIN criteria.

**Results:**

A total of 2103 articles were identified, of which 140 proceeded to full text review. A total of eight different OHRQoL measures were identified and psychometrically analysed.

**Discussion:**

The majority of measures used to assess the impact of PD and PI were not validated on local populations and were non-specific, generated by clinicians and researchers. The three condition specific measures were adaptations of the Oral Health Impact Profile, a non-specific patient reported outcome measure which may not accurately reflect the specific signs, symptoms and overall impact of PD/PI on an individual’s OHRQoL.

**Conclusion:**

There is a necessity for a condition-specific instrument to be developed to ensure that the impact of periodontal disease and peri-implantitis on OHRQoL is captured accurately.

## Introduction

Severe periodontitis is the sixth-most prevalent health condition globally, affecting nearly 750 million people worldwide [1]. The immune-mediated, inflammatory response that develops in the periodontium (the surrounding structures of the teeth) can lead to the destruction of connective tissue and bone, which can ultimately lead to tooth loss [2]. The physical and psychosocial effects of periodontal disease can significantly impact a patient’s oral health-related quality of life (OHRQoL) and have been the subject of multiple systematic reviews [3–5]. OHRQoL is a multi-dimensional construct that incorporates not only physical aspects such as function and oral health, but also considers social and emotional factors [6].

Patient-reported outcome measures (PROMs) are standardised questionnaires designed to measure an individual’s perception of their health outcomes. Such measures may incorporate the signs and symptoms of a disease, functional status and health-related quality of life [7]. Patients with experience of a disease are best placed to assess its impact on quality of life, and so their input into the development and validation of PROMS is crucial [8, 9]. There are a range of PROMs available to capture the impact of oral health conditions on OHRQoL that are utilised in both clinical and research settings [10–12].

There are a broad range of criteria that are recommended in the optimal development and validation of a PROM. Validation of a PROM ensures that it actually measures what it is intended to measure. The Consensus-base Standards for the Selection of Health Measurement Instruments (COSMIN) checklist is a tool developed to assess the methodological quality of studies that utilised the measurement properties of PROMs, the components of which were postulated in 2007 [13]. It also aims to inform evidence-based instrument selection [14]. The Study Design Checklist has been updated and adapted on several occasions [15]. Broadly, eight categories were proposed to analyse PROMs: content validity; internal consistency; criterion validity; construct validity; reproducibility; responsiveness; floor and ceiling effects and interpretability. The definitions of each can be found in Supplementary Table 1 [13, 15]. The checklist also aims to inform evidence-based instrument selection [13]. The use of an inappropriate instrument may have detrimental consequences to outcome measurement, such as over or underestimating the impact on quality of life.

To the authors’ knowledge, to date there have been no published attempts to identify the range of instruments used in the assessment of OHRQoL in patients with periodontal disease and peri-implantitis. Furthermore, these instruments have not previously undergone rigorous assessment against the criteria outlined in the COSMIN Study Design Checklist. As such, it remains unclear what the range of instruments currently being used in periodontal OHRQoL research is, and hence what the necessity for a core outcome set is. Moreover, it is uncertain whether instruments currently being used have been designed optimally to capture patient-reported impacts in this field.

Consequently, the aims of this review are twofold:

1. To systematically review the available literature to identify the range of instruments used to assess the impact of periodontitis and peri-implantitis on OHRQoL in adults.
2. To determine the quality of these instruments using previously established quality criteria.

An adaptation of the COSMIN criteria was applied to the studies that proceeded to further analysis [13]. This decision was based upon a similar article within the field of OHRQoL research [16]. To the authors’ knowledge, this is the only other article within this field that applies quality criteria to OHRQoL measures. The quality criteria that were applied was an adapted version of the COSMIN methodology for systematic reviews of Patient‐Reported Outcome Measures [13].

## Materials and methods

The systematic review protocol was prospectively registered on the Open Science Framework on 31^st^ October 2022, which can be found in the Supplementary Material.

### Eligibility criteria

An article was deemed eligible if it was available in English language; examined the relationship between oral health-related quality of life and periodontal disease or peri-implantitis; was conducted on an adult population (18 years of age or older) and reported primary data.

### Search strategy

Four separate databases (Scopus, Web of Science, Medline and Embase) were used to generate results. Multiple MeSH terms were utilised for OHRQoL, periodontitis, peri-implantitis and surveys. Articles published up until October 2022 were considered. An example of the search strategy can be found as Supplementary Material.

Search terms included the terms “oral health related quality of life”, “periodontal disease”, “periodontitis”, “peri-implantitis”, “questionnaire”, “survey” and all relevant MeSH terms. The search was originally conducted on 31^st^ October 2022.

### Study selection

Title screening was undertaken by one author (JJ) following removal of duplicates. Abstract screening was then undertaken by two reviewers (JJ and RH), with any disagreement resolved through discussion. All remaining articles underwent full text screening by two authors (JJ and JH) independently and data extraction was then undertaken for all papers meeting the inclusion criteria. These authors extracted data from 10 randomly selected articles for training and calibration purposes to ensure consistency prior to completing data extraction for the remainder of the papers. The data was inputted via Microsoft® Excel® (Microsoft Corporation, Washington, United States) If the required data were missing or unclear, this was highlighted as such within the data extraction form and reflected upon as part of the subsequent analysis.

### Analysis

Narrative synthesis of characteristics of all included studies assessing OHRQoL in patients with periodontitis and peri-implantitis was undertaken. Instruments that appeared within the review on more than five occasions or were condition-specific proceeded to further analysis against the COSMIN criteria. These criteria attribute an overall positive, negative or indeterminate rating to each measure.

The authors considered that within the range of OHRQoL instruments being used in these studies, it was expected that the majority would be generic (i.e. suitable for use across a broad range of conditions) though a small minority could be condition-specific (i.e. created to assess OHRQoL only in a specific condition). Whilst there are advantages in the use of generic OHRQoL measures such as comparing the impact of one disease to another, the specificity of the signs and symptoms of PD/PI may not be accurately captured with generic measures, making this a somewhat crude comparison.

## Results

**Fig. 1:**
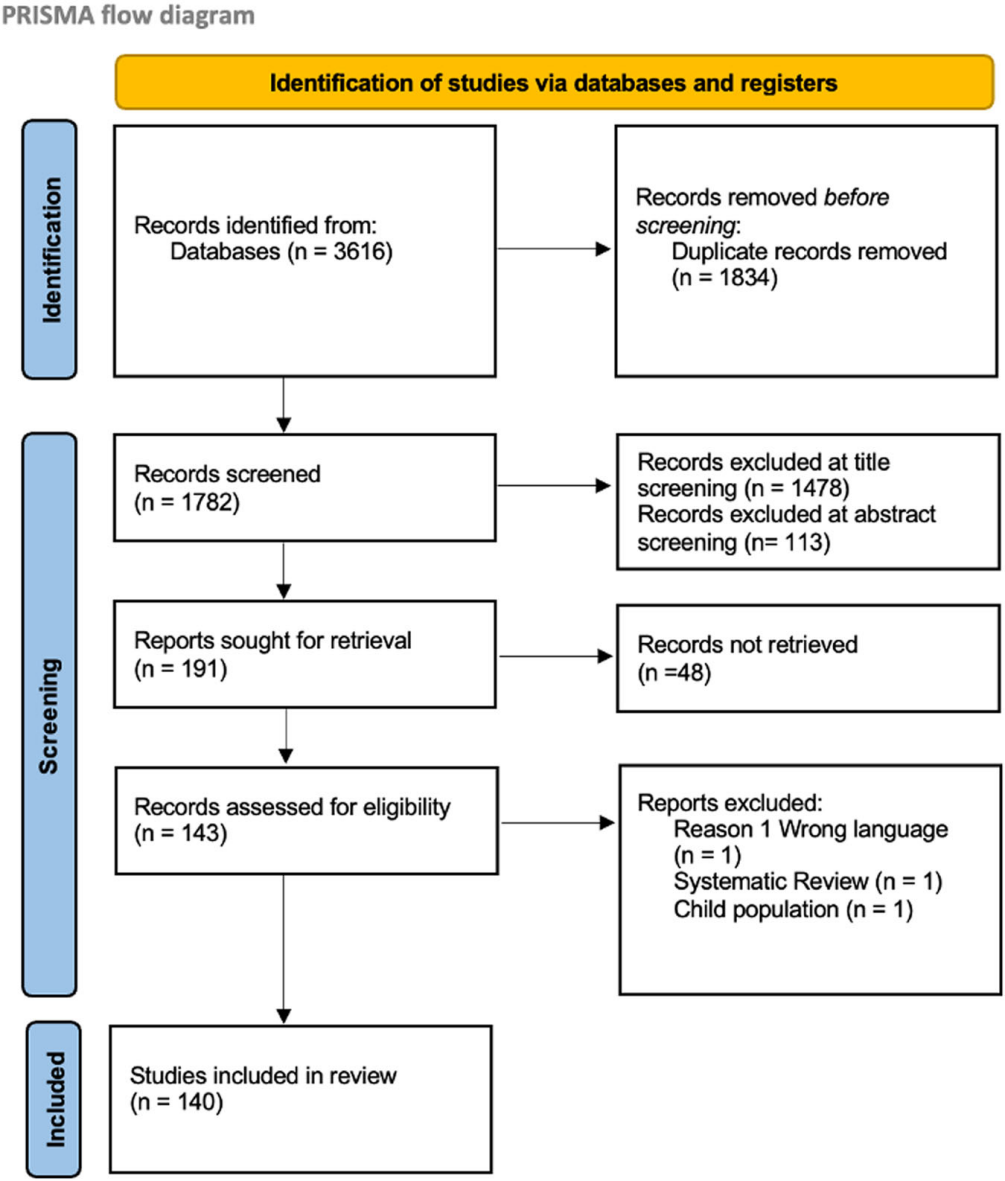
PRISMA flow diagram.

From an initial 2103 articles, 140 studies proceeded to data extraction following the removal of duplicates and screening (Fig. 1).

The majority of studies used a variation of the Oral Health Impact Profile (*n* = 110). Several studies used the OHRQoL-UK (*n* = 15). Most of the studies (*n* = 103) were either fully or in part conducted in a specialist hospital or academic setting, with 16 studies involving patients in a general practice setting. The majority of articles reported that both periodontitis and peri-implantitis affected OHRQoL. Only six articles examined the relationship between oral health-related quality of life and peri-implantitis.

Studies included within the review included a broad range of ages of participants, from 18 years in one study to 103 years in another. Diagnoses varied from periodontal health to severe periodontitis. The majority of studies (*n* = 112) did not specify race or ethnicity of participants and so the relationship between this variable and oral health-related quality of life could not be investigated.

Two condition-specific measures were identified for periodontal disease and one for peri-implantitis. Both the Oral Health Impact Profile for Chronic Periodontitis [17] and the Oral Health Impact Profile applied to Periodontal Disease (OHIP-14-PD) [18] were adapted versions of the Oral Health Impact Profile devised by Slade and Spencer some years earlier [19]. All three measures lacked the involvement of stakeholders during development.

The majority of studies (*n* = 74) allowed patients to self-administer the questionnaires, whilst 38 studies involved interviewer-administered questionnaires. The remaining 28 studies did not state the mode of administration. It is generally considered preferable for individuals to complete questionnaires themselves rather than through a clinician or researcher. Clinician-centric biases can result in errors in recording data to match the expected outcomes (‘observer bias’) and can lead to interviewers subconsciously obtaining answers that support preconceived notions (‘interviewer bias’) [20].

The majority of studies identified were classified as cross-sectional in design (*n* = 82) with 17 cohort studies, 12 case-control studies and 9 randomised controlled-trials.

The following measures were analysed against the proposed criteria:

- Multiple versions of the Oral Health Impact Profile (OHIP).
- OHQoL-UK.
- The Oral Impact on Daily Performance (OIDP).
- The Oral Health-related quality of life model for Dental Hygiene (OHRQL).
- The Geriatric Oral Health Assessment Index (GOHAI).
- The Oral Health Impact Profile applied to Periodontal Disease (OHIP-14-PD).
- The Oral Health Impact Profile for Chronic Periodontitis (OHIP-CP).
- The Oral Health Impact Profile for Dental Implants.

The development and application of each instrument is summarised below for context, and the results of the COSMIN analysis are shown in Supplementary Table 2. For measures deemed specific to periodontal disease or peri-implantitis, further details on their development and the psychometric properties that were investigated have been analysed and detailed below. Definitions for criterion are described in Table 1.

**Table 1:** Definitions of criteria applied to measures.

Content validity	Internal consistency	Criterion validity	Construct validity	Reproducibility	Responsiveness	Floor and ceiling effects	Interpretability
Clear description of measurement aim, target population, concepts measured and whether target population involved in item selection	Cronbach’s alpha <0.70 and 0.95	Correlation with gold standard ≥ 0.70	Specific hypotheses formulated, 75% results in accordance with these hypotheses	A) Agreement: MIC < SDC or MIC outside the LOA or convincing argument that agreement is acceptable B) Reliability: ICC/weighted kapa ≥0.70	SDC or SDC < MIC or MIC outside the LOA or RR > 1.96	≤ 15% respondents achieved the highest or lowest possible scores	Mean and SD scores presented of at least four relevant subgroups of patients AND MIC defined

### Generic OHRQoL instruments frequently used in periodontal/peri-implantitis research

#### The Oral Health Impact Profile (OHIP-49)

The Oral Health Impact Profile was published in 1994, and coined OHIP-49 [19]. Conceptual domains to be included in the measure were derived from Locker’s earlier model of oral health [21]. To assess the social impact of the domains, 535 statements were obtained from 64 dental patient interviews as part of a cross-sectional study of older adults in South Australia, who were recruited from two dental hospital clinics and a private dental office. From this, 49 statements were obtained from the patient group, which were subsequently weighted for perceived importance and validated. A shorter version with only fourteen items (OHIP-14) was created some years later [22].

#### OHIP-14

Having identified the necessity for a more succinct instrument to assess OHRQoL, Slade derived and validated a subset of OHIP-49 in 1997 [22]. Described as the short-form oral health impact profile, OHIP-14 consisted of 14 items that, similarly to OHIP-49, captured 7 dimensions described within Locker’s conceptual model of oral health: functional limitation, physical pain, psychological discomfort, physical disability, psychological disability, social disability and handicap [21]. Items were proposed by eliminating items that previously applied only to denture wearers and items where 5% or more responses were left blank or marked “don’t know” within the original cross-sectional study of older adults in South Australia [23]. Statistical tests were used to derive questions that could capture information from the original OHIP-49 instrument. Participants were not consulted as to which items were included within the OHIP-14 instrument, despite them being best placed to assess their own quality of life [9].

#### OHQOL-UK

OHQoL-UK was developed following open-ended interviews with a random probability sample of 1865 United Kingdom residents, which were subsequently coded for analysis. From this, 16 key areas of oral health related quality of life were identified and proposed as part of the measure [24]. Further interviews were conducted with 500 participants whereby the new measure was administered and validated. Respondents rate the ‘impact’ of each ‘effect’ which acts as a weighting score. It has been suggested elsewhere that weighting does not improve the psychometric performance of a measure [25, 26]. An overall score between 16 to 144 can therefore be generated, with a lower score indicating a poorer OHRQoL [27].

#### Geriatric Oral Health Assessment Index (GOHAI)

The Geriatric Oral Health Assessment Index is a 12-item questionnaire whereby participants state the frequency of which they experience each item. The questionnaire uses a Likert scale ranging from always to never. Total scores for an individual can range from 0 to 48, with a higher score indicating a poorer OHRQoL.

The original study was published in 1990 following initial pilot testing on a convenience sample of 87 older adults. Participants were based in Los Angeles and had a mean age of 76 years. A large-scale field test was conducted with 1755 participants with a mean age of 74 years, 57% female, 87% white and 61% married [28]. The generation of the initial framework for the measure was based upon a literature review. Additional items were generated through consultation with health care providers and questionnaires performed in a hospital dental clinic. It could therefore be suggested that limited patient and public involvement was incorporated into the development of the GOHAI. In comparison, four studies using the GOHAI had mean ages of 46, 44, 51 and 51 years respectively, with one study including a patient aged 21 years. The original validated measure was not designed for such age ranges, questioning its suitability amongst younger cohorts.

#### Oral Health Related Quality of Life Model for Dental Hygiene (OHRQL)

The OHRQL model was developed in 1998 in the USA [29]. The instrument separated 22 questions into seven subscales: pain, dry mouth, eating/chewing function, speech function, social function psychological function and oral health perception. OHRQL was based upon three pre-existing models: The Wilson & Clearly HRQL model, the Natural History of Disease Model and Neuman’s Systems Model for Nursing.

#### Oral Impacts on Daily Performances (OIDP)

The OIDP measures the impact to which an individual’s ability to perform physical, psychological and social activities is compromised due to poor oral health. The original 9 items were based upon the WHO’s International Classification of Impairments, Disabilities and Handicaps amended for dentistry. One item was later considered redundant and excluded [30]. OIDP scores were calculated by multiplying frequency and severity scores of daily performances, which accommodated for weighting. Later versions have been applied without weighting for simplicity and similar measures have shown to be appropriate and significant without weighting applied [30, 31]. Patients were not actively involved in the selection of items.

### Condition-specific instruments used in periodontal/peri-implantitis research

#### Oral Health Impact Profile for Chronic Periodontitis (OHIP-CP)

The first attempt to develop and validate an OHRQoL measure specific to periodontal disease was published in 2017 [17]. A study sample of 420 patients referred to a periodontal clinic of a Chinese hospital were included. The sample size was determined following exploratory factor analysis. Items were generated following a literature review focusing on OHIP-49. Of the 23 items proposed, 21 items were derived from OHIP-49 with the addition of 2 items referencing bleeding gums and loosening teeth. Items were deleted following qualitative content analysis and quantitative test theory resulting in 18 items remaining in the final instrument.

#### Psychometric properties

Content validity was determined through involvement of patients, investigators and an independent expert panel, however the methodology and involvement of such groups remains unclear. The expert panel consisted of periodontal professionals and psychologists. Construct validity was assessed through ‘exploratory factor analysis’. Discriminative ability was examined by comparing mean score differences in severity of disease. A standard global rating of oral health was included to determine ‘convergent validity’ of the proposed instrument. With respect to internal consistency, the instrument for the whole scale produced Cronbach’s alpha of 0.936, which scores positively with respect to the criteria proposed by Terwee et al. [13]. Moreover, reproducibility was evaluated through calculating intraclass correlation coefficients (ICCs). A total of 30 patients completed the OHIP-CP again after 2 weeks to support this, which is an appropriate time period according to the proposed criteria. ICC values were between 0.805 (95% CI = 0.578–0.898) and 0.887 (95% 0.679–0.979). The Krushkal-Wallis test was performed to examine the instruments discriminative validity.

#### Oral Health Impact Profile applied to Periodontal Disease (OHIP-14-PD)

The OHIP-14-PD is an adaptation of OHIP-14 (Spanish version) to evaluate the impact of periodontal disease. OHIP-14-PD was validated in 2018 in Mexico [32].

### Psychometric properties

Content validity was established through ‘expert judgement’ – a panel consisting of periodontal and public health experts. Lack of target population involvement at this stage scores negatively with respect to the criteria proposed by Terwee et al. who advocate clear description of measurement aim alongside the target population being involved in item selection. OHIP-14 items were adjusted in accordance with the main signs and symptoms associated with periodontal disease according to the American Academy of Periodontology classification system. No thorough statistical analysis was conducted to support the internal consistency of the instrument, cross-cultural adaptivity or validity.

#### Oral Health Impact Profile for Dental Implants

One study conducted in Bulgaria used a modified version of the OHIP-20 for Dental Implants. The measure was translated into Bulgarian by clinicians. This is in contrast to gold standard guidelines, which highlights the necessity of both forwards and backwards translations [33]. Internal consistency was measured through Cronbach’s alpha of 0.858, scoring positively. The measure was not validated on the population being investigated, nor with any other cohort to the authors’ knowledge. Despite being condition specific, this PROM is not currently suitable nor validated for use.

### Psychometric properties of instruments

The results of the analysis of each of the measures detailed above against the COSMIN quality criteria are shown in Supplementary Table 2 [13]. It is the opinion of the authors that the presence of four subgroups was not always deemed appropriate for patients with periodontal disease or peri-implantitis, as the articles were highly heterogeneous, focusing on completely different variables and clinical parameters. A large number of studies utilising OHIP-14 contained no psychometric analysis other than the presence of Mean and SD values. Therefore, only studies that contained analysis beyond Mean/SD were included for the OHIP-14 data. Studies utilising OHIP-14 that had only mean/SD values are not included in the table.

## Discussion

To the authors’ knowledge, this study describes the first published systematic review exploring the use of patient-reported outcome instruments in periodontal and peri-implantitis research. The majority of studies demonstrated how these two conditions have an impact on OHRQoL, which is consistent with findings from other reviews [3–5, 34]. A wide range of instruments were used across the identified studies, though the majority used a variant of the generic OHIP instrument. Only three condition-specific instruments were identified from the included studies. All of the instruments that underwent analysis against the COSMIN criteria, including the OHIP instruments, were found to have deficiencies in their psychometric properties, which calls into question the suitability of these instruments for research in this field.

All of the three condition-specific instruments that were identified through this study were adaptations of an original OHIP instrument. As such, they may inherit the deficiencies of the original instrument and require validation in their own right. Whilst the adaptation of existing generic instruments for use in condition-, or population-specific contexts is increasingly common (such as OHIP-TMD and Child Oral Health Impact Profile [35, 36]), there are some advantages to developing a new instrument de novo, as it grants the opportunity to comply with modern best-practice guidance and can facilitate active involvement of stakeholders from the outset.

The majority of the included studies were conducted in secondary or tertiary care settings. This is interesting, given that the majority of periodontal conditions are managed in primary care settings. There are a number of challenges to conducting research in a primary care setting, such as increasing time pressure on service provision as well as indemnity and legislative challenges. Despite this, there is a real need for primary care-based research, as the population of patients attending for care in this setting may have notable differences to those seeking care in a secondary care setting. This study also highlighted a lack of longitudinal research in this field. Whilst cross-sectional studies can provide a useful start for the validation process for PROMs, longitudinal studies provide an opportunity to explore changes in OHRQoL over time, and the sensitivity of an instrument to capture this.

There were a notable lack of studies exploring peri-implantitis and OHRQoL. This may be reflective of the relative prevalence of those with peri-implantitis in comparison to patients exhibiting periodontitis. Peri-implantitis is also a relatively new condition meaning research in this field is still developing.

In addition to the absence of patient involvement in the development of the measures, the most common deficiencies in the psychometric properties of these instruments were a lack of criterion validity; construct validity; absence of testing for responsiveness as well as a lack of acknowledgement of floor/ceiling effects. There was a notable lack of cross-cultural adaptation across the majority of the instruments, particularly amongst countries with the largest variation in patient demographics.

This study has a number of strengths, including the use of the COSMIN criteria, a highly regarded tool for appraising the psychometric properties of these instruments. The COSMIN initiative is a multidisciplinary team with expertise in developing and evaluating outcome measurement instruments [37]. To the authors’ knowledge, this is the first time these criteria have been applied to periodontal research. There are some limitations to this research. Given the high number of studies identified from the systematic review, a considerable amount of researcher time was required for data extraction which has led to an extend period between the search and manuscript submission.

When considering the deficiencies in the quality of existing generic and condition-specific PROMs identified in this study, their ongoing use in periodontal and peri-implantitis research can be called into question. There is a need for a condition-specific instrument to be developed, with involvement of patients and the public from an early stage, consideration of psychometric properties throughout development, and longitudinal validation with an appropriate population. A condition specific measure that can accurately assess the impact of periodontal disease would have a large array of applications. This may be on a patient level, such as evaluating the impact of treatment on an individual over time as well as allowing individuals to visualise and measure the changes in their own OHRQoL, which may be a useful aid in improving compliance with treatment. In a research setting, the measure could be applied to evaluate or compare interventions, which in turn could serve a role in the commissioning and delivery of treatment to patients on a wider scale.

## Conclusion

The generic and condition-specific OHRQoL instruments used in periodontitis and peri-implantitis have a range of deficiencies in their measurement properties. There is a need for a condition-specific instrument to be developed, to ensure that the impact of periodontal disease and peri-implantitis on OHRQoL is captured accurately.

## Supplementary information

PRISMA Checklist

Supplementary Information

## Data Availability

Data available upon request to corresponding author.
