# Supplementary material for: A systematic review of the patient reported outcome measures used to assess the impact of periodontitis and peri-implantitis on oral health related quality of life
Source: BDJ Open. 2025 Mar 28;11:29. doi: 10.1038/s41405-024-00273-w (PMC11953381; doi:10.1038/s41405-024-00273-w)
Supplement: Supplementary file 2 — Supplementary Information [file 41405_2024_273_MOESM2_ESM.pdf]

Supplementary Material

Registration of Systematic Review with Open Science Framework

https://osf.io/647vb (accessed June 2024).

SI Table 1 – Reproduction of the definitions of quality criteria proposed for measurement properties of health status questionnaires [13]

Property	Definition
**Content Validity**	The extent to which the domain of interest in comprehensively sampled by the item in question
**Internal consistency**	The extent to which items in a (sub)scale are intercorrelated, thus measuring the same construct
**Criterion validity**	The extent to which scores on a particular questionnaire relate to a gold standard
**Construct validity**	The extent to which scores on a particular questionnaire relate to other measures in a manner that is consistent with theoretically derived hypotheses concerning the concepts that are being measured
**Reproducibility**	
Agreement	The extent to which the scores on a repeated measures are close to each other
Reliability	The extent to which patients can be distinguished from each other, despite measurement errors
**Responsiveness**	The ability of a questionnaire to detect clinically
**Floor and ceiling effects**	The number of respondents who achieved the lowest possible or the highest possible score
**Interpretability**	The degree to which one can assign qualitative meaning to quantitative scores.

Example Search Strategy

1.	"Quality of Life"/
2.	OHRQoL.mp.
3.	QoL.mp.
4.	Quality of life.mp.
5.	1 or 2 or 3 or 4
6.	Oral Health/
7.	oral health.mp.
8.	6 or 7
9.	exp Periodontal Diseases/
10.	gum disease*.mp.
11.	peri-implantitis.mp.
12.	periodontal disease*.mp.
13.	periodontitis.mp.
14.	9 or 10 or 11 or 12 or 13
15.	5 and 8 and 14
16.	impact.mp.
17.	measure*.mp.
18.	tool*.mp.
19.	exp "Surveys and Questionnaires"/
20.	survey*.mp.
21.	questionnaire*.mp.
22.	oral health impact profile.mp.
23.	OHIP-14.mp.
24.	OHIP-49.mp.
25.	OHQoL-UK.mp.
26.	quality assessment tool/
27.	16 or 17 or 18 or 19 or 20 or 21 or 22 or 23 or 24 or 25
28.	5 and 8 and 14 and 27

SI Table 2: Psychometric analysis of OHRQoL measures used in patients with periodontal disease and peri-implantitis

Version of measure	No. of studies	Content validity	Internal consistency	Criterion validity	Construct validity	Reproducibility	Responsiveness	Floor and ceiling effects	Interpretability
		Clear description of measurement aim, target population, concepts measured and whether target population involved in item selection	Cronbach's alpha <0.70 and 0.95	Correlation with gold standard ≥ 0.70	Specific hypotheses formulated, 75% results in accordance with these hypotheses	A) Agreement: MIC < SDC or MIC outside the LOA or convincing argument that agreement is acceptable<br>B) Reliability: ICC/weighted kapa ≥0.70	SDC or SDC < MIC or MIC outside the LOA or RR > 1.96	≤ 15% respondents achieved the highest or lowest possible scores	Mean and SD scores presented of at least four relevant subgroups of patients AND MIC defined
**OHIP-49**									
**Original**	2		Cronbach's alpha 0.97 (possible redundancy of items)						
**European**	1								
**German**	2								MIC defined in one study, mean and SD defined in both studies
**Malayalam**	1								
**OHIP-14**									
**Version of measure**	**No. of studies**	**Content validity**	**Internal consistency**	**Criterion validity**	**Construct validity**	**Reproducibility**	**Responsiveness**	**Floor and ceiling effects**	**Interpretability**
**Original (Australian)**	3				Significant direct effects				MID defined (1 study), Mean/SD

					between self-esteem, self-efficacy and OHRQoL				presented (3 studies)
**Turkish**	3		Cronbach's alpha 0.73-0.88						Mean/SD presented (3 studies).
**Brazilian**	6		Cronbach's alpha 0.79-0.92 (6 studies)			ICC 0.82 (2 studies). Repeated administration in 10% sample 7-day interval (3 studies)		Floor and ceiling effects not observed (2 studies)	MID defined (2 studies) mean and SD presented (5 studies)
**Arabic**	1		Cronbach's alpha 0.89			Test-retest correlation 0.85-0.97 (MIC, ICC/weighted Kappa not stated)			Mean/SD presented
**Spanish**	1		Cronbach's alpha 0.76						Mean/SD presented
**Taiwanese**	2		Cronbach's alpha 0.9-0.95						Mean/SD presented (1 study)
**Italian**	1		Cronbach's alpha 0.853						Mean/SD presented
**Persian**	1		Cronbach's alpha 92.4-96.2						Mean/SD presented
**Chinese**	4		Cronbach's alpha 0.73-0.94 (3 studies)		Scores significantly correlated with perceived oral health status, impact of oral health				MID defined in 1 study, mean/SD presented (3 studies)

					on daily life and dental treatment need				
**Norwegian**	1		Cronbach's alpha 0.89						Mean/SD presented
**Romanian**	1		Cronbach's alpha 0.88						Mean/SD presented
**Swedish**	1		Cronbach's alpha 0.92-0.95						Mean/SD presented
**Malaysian**	1		? Cronbach's alpha 0.95 quoted from previous study, not calculated						Mean/SD presented
**German**	1								MID stated based upon previous study, Mean/SD presented
**Indian**	1					Reliability Cohen's kappa 0.84-0.87			Mean/SD presented
**French**	1							Floor effect observed	
**Unknown (in Sweden)**	1		Cronbach's alpha 0.87			Weighted kappa 0.15-0.72			
**OHQOL-UK**									
**Version of measure**	**No. of studies**	**Content validity**	**Internal consistency**	**Criterion validity**	**Construct validity**	**Reproducibility**	**Responsiveness**	**Floor and ceiling effects**	**Interpretability**
**Original**	3		Cronbach's alpha 0.96						Mean/SD available (2 studies), no MIC

**Brazilian-Portuguese [ref Dini]**	4		Cronbach's alpha 0.97 and 0.89 (2 studies)			Test re-test at 7-day interval with 20 individuals (2 studies). Spearman's rank correlation coefficient 0.89			Mean/SD available (2 studies), no MIC
**Telugu**	1								Mean/SD available, no MIC
**Turkish**	3								Mean/SD available (2 studies), no MIC
**Persian**	1		Cronbach's reliability coefficient 0.66-0.77						Mean/SD available, no MIC
**Original (used on Swedish population – language unclear)**	1		Cronbach's alpha 0.96	Comparison to global self-reported oral health					MIC defined with mean and SDs evident
**Malay**	1								Mean/SD available, no MIC
**Indian 'local regional language' (not stated)**	1								Mean/SD available, no MIC
**GOHAI**									
**Version of measure**	**No. of studies**	**Content validity**	**Internal consistency**	**Criterion validity**	**Construct validity**	**Reproducibility**	**Responsiveness**	**Floor and ceiling effects**	**Interpretability**
**Turkish**	4		Cronbach's alpha 0.83, 0.78, 0.76.						Mean/SD available in 3 studies, no MIC

			Sample size calculation						
**Persian**	1		Cronbach's alpha 76.8-85%						Mean/SD available, no MIC
**Swedish**	2		Cronbach's alpha 0.84 and 0.82-0.83				MID calculated in accordance with effect size and SEM. Mean change < SEM	Floor and ceiling effects accounted for	Mean/SD available, MIC defined in 1 study
**Brazilian-Portuguese**	1		Cronbach's alpha 0.72						Mean/SD available, no MIC
**Taiwanese**	1								
**Hindi**	1								Mean/SD available, no MIC
**USA**	1		Cronbach's alpha 0.90						
**Oral Impact on Daily Performance (OIDP)**									
**Version of measure**	**No. of studies**	**Content validity**	**Internal consistency**	**Criterion validity**	**Construct validity**	**Reproducibility**	**Responsiveness**	**Floor and ceiling effects**	**Interpretability**
**? Taiwanese**	1		Cronbach's alpha 0.90						Mean/SD available, no MIC
**Brazilian - Portuguese**	4		Cronbach's alpha 0.92 (1 study)			Re-administered after 7 days in 20 individuals			Mean/SD available, no MIC
**UK**	1								Mean/SD available, MID defined
**Oral Health Related Quality of Life Model for Dental Hygiene (OHRQL)**									
**Version of measure**	**No. of studies**	**Content validity**	**Internal consistency**	**Criterion validity**	**Construct validity**	**Reproducibility**	**Responsiveness**	**Floor and ceiling effects**	**Interpretability**

**Japanese**	3		Cronbach's alpha 0.913 (1 study)						Mean/SD available, no MIC
**Oral Health Impact Profile for Chronic Periodontitis (OHIP-CP)**									
**Version of measure**	**No. of studies**	**Content validity**	**Internal consistency**	**Criterion validity**	**Construct validity**	**Reproducibility**	**Responsiveness**	**Floor and ceiling effects**	**Interpretability**
**Chinese**	2	Questionable and limited target population involvement in item selection	Cronbach's alpha 0.936	Correlation with global oral health rating < 0.70		ICC values between 0.805 (95% CI = 0.578-0.898 and 0.887 (95% 0.679-0.979)			Mean/SD available, MID defined
**Oral Health Impact Profile applied to Periodontal Disease (OHIP-14-PD)**									
**Version of measure**	**No. of studies**	**Content validity**	**Internal consistency**	**Criterion validity**	**Construct validity**	**Reproducibility**	**Responsiveness**	**Floor and ceiling effects**	**Interpretability**
**Mexico**	2								Mean/SD available, no MID (1 study)
**Oral Health Impact Profile for Dental Implants**									
**Version of measure**	**No. of studies**	**Content validity**	**Internal consistency**	**Criterion validity**	**Construct validity**	**Reproducibility**	**Responsiveness**	**Floor and ceiling effects**	**Interpretability**
**Bulgarian**	1		Cronbach's alpha 0.858						Mean/SD available, no MID

Notes:

For articles using OHIP-14, only studies that incorporated psychometric analysis beyond the presentation of mean and standard deviations were included. OHRQoL measures within the articles were given a positive, negative or indeterminant rating in accordance with the quality criteria proposed. [13] For some articles, a positive rating was included for interpretability despite fewer than four relevant subgroups being present. MIC = Minimally important change; SDC = Smallest detectable change; LOA = limits of agreement; ICC = Intraclass correlation; SD = standard deviation.

Negative rating

Indeterminate rating

Positive rating
